# Skeletal development of the hand and wrist: digital bone age companion—a suitable alternative to the Greulich and Pyle atlas for bone age assessment?

**DOI:** 10.1007/s00256-017-2616-7

**Published:** 2017-03-25

**Authors:** Paul M. Bunch, Talissa A. Altes, Joan McIlhenny, James Patrie, Cree M. Gaskin

**Affiliations:** 1 Department of Radiology, Massachusetts General Hospital, 55 Francis Street, Boston, MA 02114 USA; 2 Department of Radiology, University of Missouri, One Hospital Drive, Columbia, MO 65212 USA; 3 Department of Radiology and Medical Imaging, University of Virginia Health System, PO Box 800170, 1215 Lee Street, Charlottesville, VA 22908 USA; 4 Department of Health Evaluation Sciences, University of Virginia Health System, PO Box 800717, Charlottesville, VA 22908 USA

**Keywords:** Children, Radiography, Bone age, Skeletal age, Skeletal maturity, Development

## Abstract

**Purpose:**

To assess reader performance and subjective workflow experience when reporting bone age studies with a digital bone age reference as compared to the Greulich and Pyle atlas (G&P). We hypothesized that pediatric radiologists would achieve equivalent results with each method while digital workflow would improve speed, experience, and reporting quality.

**Materials and methods:**

IRB approval was obtained for this HIPAA-compliant study. Two pediatric radiologists performed research interpretations of bone age studies randomized to either the digital (Digital Bone Age Companion, Oxford University Press) or G&P method, generating reports to mimic clinical workflow. Bone age standard selection, interpretation-reporting time, and user preferences were recorded. Reports were reviewed for typographical or speech recognition errors. Comparisons of agreement were conducted by way of Fisher’s exact tests. Interpretation-reporting times were analyzed on the natural logarithmic scale via a linear mixed model and transformed to the geometric mean. Subjective workflow experience was compared with an exact binomial test. Report errors were compared via a paired random permutation test.

**Results:**

There was no difference in bone age determination between atlases (*p* = 0.495). The interpretation-reporting time (*p* < 0.001) was significantly faster with the digital method. The faculty indicated preference for the digital atlas (*p* < 0.001). Signed reports had fewer errors with the digital atlas (*p* < 0.001).

**Conclusions:**

Bone age study interpretations performed with the digital method were similar to those performed with the Greulich and Pyle atlas. The digital atlas saved time, improved workflow experience, and reduced reporting errors relative to the Greulich and Pyle atlas when integrated into electronic workflow.

## Introduction

The determination of pediatric skeletal maturity is important for a number of clinical indications; for example, the diagnosis of disorders of growth and development, the timing of pediatric corrective surgeries for limb-length discrepancy and scoliosis, and the assessment of treatment response in certain endocrine conditions [1]. These assessments also have important applications in forensic science [2]. Multiple techniques for assessing skeletal maturity have been described [3–5], but the most widely accepted technique is that of Greulich and Pyle [6, 7].

Greulich and Pyle’s *Radiographic Atlas of Skeletal Development of the Hand and Wrist* (G&P) [6] contains left hand radiographs selected as sex-specific developmental standards at different ages. G&P also contains data tables of sex-specific mean skeletal ages and standard deviations at various chronological ages, from which calculations can be made to determine whether a child’s skeletal maturity is normal.

The principal components of G&P are all based upon data derived from the Brush Foundation Study of Child Growth and Development [6], in which a cohort of 999 children in Ohio were serially examined at intervals of 3 to 12 months from 1931 to 1942. A left hand radiograph was a component of each examination, and nearly 14,000 left hand radiographs were obtained during the study and ultimately painstakingly analyzed by Greulich, Pyle, and their associates during the development of G&P. Greulich and Pyle selected representative radiographs to correspond with each chronological age represented in the atlas. Comparing these chosen standards with hundreds of normal children of similar chronological age, they were then able to calculate standard deviations at each chronological age represented by their chosen standards. This rigorous approach and extensive data from repeated radiation exposure in normal children have contributed to the longevity of G&P.

The work behind G&P is truly admirable; however, the standards contained within G&P are understandably imperfect. Greulich and Pyle used their data and experience to select the best available left hand radiograph for each G&P standard. Nonetheless, as a result of the natural variability in the rate at which individual bones age, it is the case that individual bones in some of the G&P standards are more or less advanced than the age the standard is intended to represent. For example, the G&P 3 year 6 month (42 month) standard is accompanied by text on the adjacent page indicating that the standard includes a 36-month first metacarpal and a 54-month lunate [6].

G&P remains in widespread clinical use today despite being a relatively old reprint and requiring a manual process. An additional potential limitation of G&P is its applicability to modern diverse populations since it is based upon a study of a more focused patient population in the 1930s [6]. More recently, alternative atlases to G&P also containing left hand radiographic standards have been developed [8–10]; however, there is overall a relative paucity of peer-reviewed data available on the clinical utility or accuracy of such alternatives [11, 12]. No previous study has compared the most recent of these alternative atlases—*Skeletal Development of the Hand and Wrist: Digital Bone Age Companion* (DBAC) (Oxford University Press, New York)—to G&P. DBAC [8] is a commercially available software application that accompanies a hardcopy bone age reference textbook based upon G&P. The precursors of the DBAC standards came from candidate clinical digital bone age images while referencing the G&P maturity indicators. These precursor images were then digitally edited so that the developmental features of each bone of the hand and wrist on each image match those of the G&P standards. To mitigate the natural variability in maturity among individual bones of the hand and wrist described in the preceding paragraph, some of the individual bones in the DBAC standards were intentionally edited to be advanced or delayed relative to their G&P counterparts to better represent the overall skeletal age of the standard as indicated by the text of G&P [8].

**Fig. 1:**
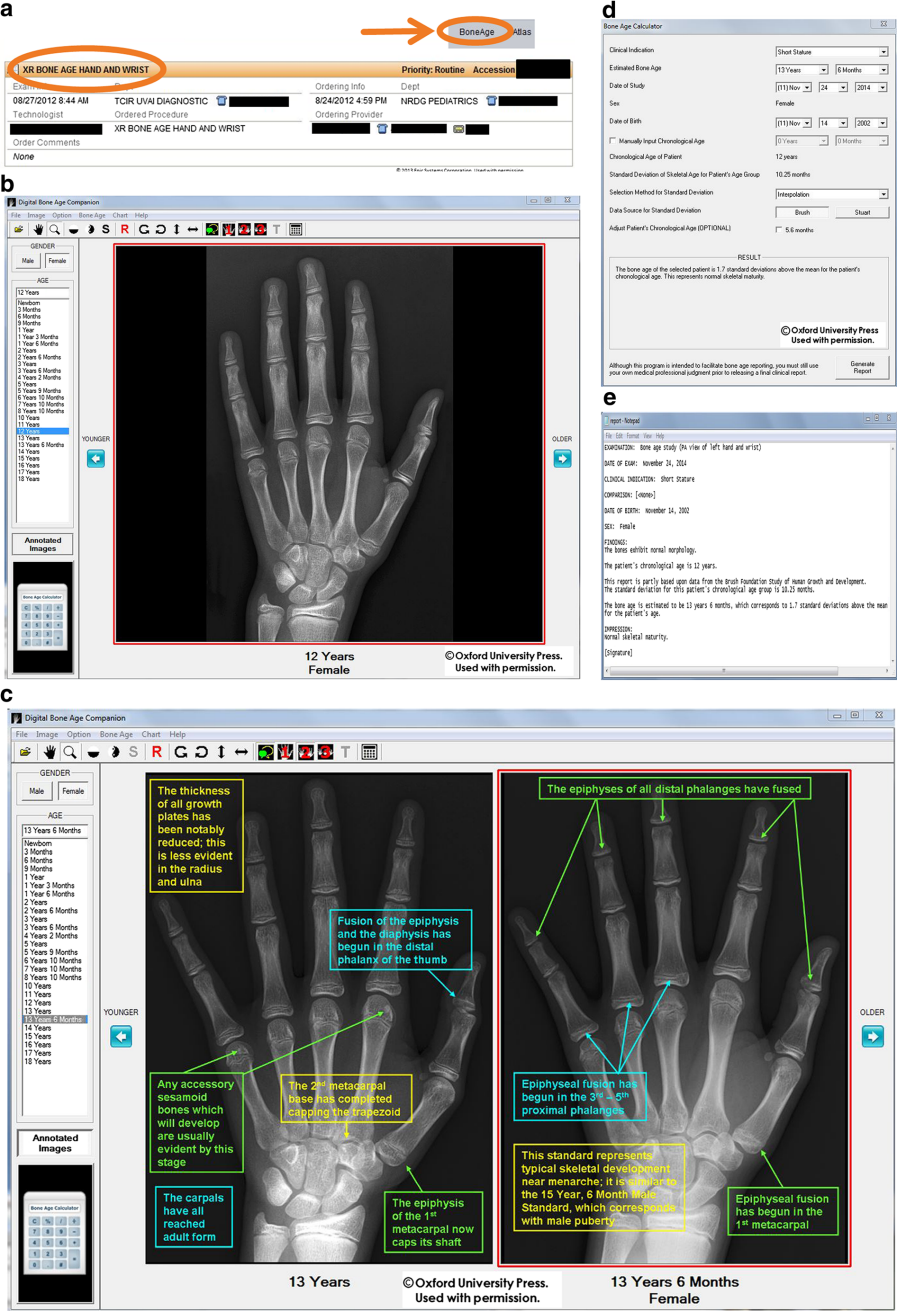
Demonstration of bone age study interpretation with the digital method integrated into clinical workflow. (The display of patient information in the software is from a hypothetical patient for illustrative purposes. Any resemblance to that of an actual patient is coincident.) Figure 1b–e from Skeletal Development of the Hand and Wrist: Digital Bone Age Companion by Gaskin et al, 2011. By permission of Oxford University Press. **a** Screenshot from RIS-EHR demonstrates anonymized bone age study requisition and “BoneAge” button (orange *arrow*) in the graphical user interface. Clicking the button launches bone age software and initiates XML file drop containing the patient’s date of birth, date of study, and gender. **b** Screenshot of bone age software. The software references the XML file and then displays the bone age standard closest to the patient’s age and matching the patient’s gender for the most likely match in the typical patient. Users may zoom in on skeletal features and adjust the window level and width to their preference. **c** Screenshot of bone age software demonstrates optional annotated standards to aid the interpreter in choosing the best match. Up to three standards (only two shown) may be reviewed side-by-side to further aid decision making. Clicking the calculator button (calculator icon at bottom left) sends the patient’s date of birth, date of study, gender, and chosen standard to the calculator. **d** Screenshot of bone age calculator. The calculator uses the correct standard deviation value to perform the skeletal maturity calculation. The software includes settings that allow for adjustments in how the calculation is performed to accommodate regional practice differences. The user can manually edit the estimated skeletal age if it falls between two standards. Clicking the “Generate Report” button (bottom right) creates a structured report that can be copy/pasted to the reporting system for final review and signature. **e** Example of structured report generated by bone age software based upon user-chosen bone age standard and automatically imported patient demographics from the RIS-EHR or PACS

The manual nature of the G&P method requires reviewing images and text within a book, looking up data in a chart, and making basic calculations (i.e., to determine if the estimated skeletal age is within 2 standard deviations at the given chronological age), which relatively slows diagnostic workflow and introduces the possibility of both observer and mathematical errors. This manual process can be even more challenging for trainees or infrequent users in general practice. DBAC attempts to modernize the approach of G&P by offering potential workflow enhancements, such as patient context sharing from PACS (picture archive and communication system) or RIS (radiology information system) to DBAC, an automated bone age calculator and report generator, as well as side-by-side standard comparisons with annotations highlighting discriminating features (Fig. 1).

At the time our study was initiated, DBAC was newly integrated into our radiology department’s clinical workflow, making it both practical and relevant for us to determine if this newer atlas provided suitable replacement to our long-standing use of G&P. The purpose of our study was to test our hypotheses that (1) pediatric radiologists would achieve equivalent bone age results when interpreting and reporting bone age studies with DBAC versus G&P and (2) digital workflow enhancements would improve speed, subjective workflow experience, and reporting quality.

## Materials and methods

This study is Health Insurance Portability and Accountability Act compliant and institutional review board approved. A waiver of informed consent was issued by the institutional review board.

### Subjects (bone age study selection)

We identified bone age examinations interpreted clinically with the G&P atlas over 1 year prior to the start of our study to limit potential for recall bias. We collected information from the reports of these bone age examinations stored on the PACS to include: (1) patient gender, (2) interpreting physician, and (3) original bone age report qualitative impression (i.e., normal vs. delayed vs. advanced skeletal maturity). The radiographs themselves were not reviewed as part of study selection. We identified 96 bone age examinations (Table 1), which had been previously interpreted at least 1 year previously at a tertiary care center by two fellowship-trained faculty pediatric radiologists (10 and 29 years of focused pediatric radiology experience) using the G&P method. The cohort was chosen to achieve an approximately even distribution by (1) patient gender and (2) interpreting physician. One-third of studies in the cohort were purposefully chosen to have abnormal skeletal maturity to create a more rigorous study more likely to reveal a difference in performance. The pediatric radiologist readers were blinded to this information.

**Table 1:** Characteristics of the children included in the bone age sample for interpretation as a group and by reader

	Gender		Chronological age (years)					Original bone age report impression		
	Male	Female	Mean	SD	Median	Min	Max	Normal	Advanced	Delayed
Total (*n* = 96)	50 (52%)	46 (48%)	10.9	4.0	11.3	1.8	17.2	63 (66%)	17 (18%)	16 (17%)
Reader 1 (*n* = 50)	24 (48%)	26 (52%)	11.1	3.9	11.5	3.7	17.2	36 (72%)	9 (18%)	5 (10%)
Reader 2 (*n* = 46)	26 (56.5%)	20 (43.5%)	10.6	4.1	10.9	1.8	15.9	27 (59%)	8 (17%)	11 (24%)

The chronological age of patients (*n* = 96) utilized in this study ranged from 1.8–17.2 years (median 11.3 years; SD 4.0 years). Additional details about the cohort are provided in Table 1.

### Bone age interpretation

The same pediatric radiologists who had previously interpreted the selected examinations for clinical purposes with the G&P atlas were blinded with respect to the original report and asked to re-read their own prior studies (*n* = 96, comprised of 50 studies interpreted by radiologist 1 and 46 by radiologist 2). Recall bias was limited by use of studies that were greater than 1 year old. Research study reads were randomized to either G&P (*n* = 48, comprised of 25 for radiologist 1 and 23 for radiologist 2) or DBAC (*n* = 48, comprised of 25 for radiologist 1 and 23 for radiologist 2). Bone age standard selection, overall impression (i.e. normal, delayed, or advanced maturity), interpretation-report cycle time, and report typographical or speech recognition errors (i.e., one error = single instance of incorrect word or spelling) were recorded for each of the research interpretations.

As the G&P method has been widely utilized for decades, it is presumed that this clinical workflow is understood. As the integrated DBAC method is likely unfamiliar to many readers, it is illustrated in Fig. 1 and described briefly here. When interpreting a bone age study, the radiologist launches DBAC by clicking on an icon within the RIS or PACS depending on the local system configuration. In our system, readers can click on an icon in either the RIS (Radiant, Epic Systems, Verona, WI) or the PACS (Carestream version 11.3, Carestream Health, Rochester, NY), choosing whichever is found to be more convenient. This same click also passes the patient and study context to DBAC, which then displays the standard of correct gender and most closely matching age. The radiologist may optionally call up additional standards and/or guiding annotations if desired until the best match is made. If the reader believes the clinical image falls between two standards, this adjustment can be manually entered into DBAC. With context sharing and standard matching complete, a structured report is then generated.

We wish to clarify our rationale for evaluating the overall skeletal maturity assessment (i.e., normal, delayed or advanced) beyond simply evaluating the bone age standard assessment. The bone age standard assessment reflects how closely the reader believes the candidate image matches one standard over another; however, the overall skeletal maturity assessment includes additional steps of looking up the standard deviation, considering the chronological age of the patient and potentially making a calculation (e.g., when the estimated skeletal age does not match the chronological age, the radiologist may compare the two to determine if they are within two standard deviations of each other), and finally classifying the study into one of three basic categories (i.e. normal, delayed, or advanced maturity). Because the overall skeletal maturity assessment includes additional steps, these are further potential sources of human error beyond performing skeletal age assessment alone. Since DBAC automates standard deviation data look-up, calculates the number of standard deviations difference between estimated skeletal age and chronological age, and classifies the numerical result (normal vs. delayed vs. advanced), there is potential for differences in overall skeletal maturity assessment between DBAC and G&P methods, even in cases with matching bone age standard assessments.

### Benchmark reading

One of our objectives was to determine whether DBAC would yield similar bone age results to G&P; however, we recognized that if we only compared DBAC-based interpretations to the original G&P-based interpretations a confounding variable would be intraobserver variability known to be intrinsic to the G&P method [2]. Thus, we desired a stronger benchmark than the original clinical interpretation alone. Since there is no perfect gold standard in the case of bone age results, we decided to use a two-out-of-three approach to establishing a benchmark with at least one of the two matching results coming from G&P. When the research interpretation agreed with the original clinical G&P-based interpretation, the two matching results were considered the benchmark. When a research interpretation disagreed with the original clinical G&P interpretation, the radiologist that produced the discrepant result between her own readings was informed of a conflict and asked to perform a blinded third reading using G&P. This generated a two-out-of-three tie-breaker result and established the benchmark. In such cases, the tie-breaking interpretation took place 4 weeks after the initial research interpretation to limit recall bias.

We wish to further clarify the rationale for using a third tie-breaking interpretation with G&P by the same reader as the benchmark. The original clinical reading alone was considered insufficient as a benchmark because of the expected intraobserver variability. An alternative study design could utilize a second reader as the tie-breaker, but that introduces interobserver variability, and our study was focused on intraobserver performance when using two different bone age methods. It should be emphasized that in all cases of discrepancy, at least one if not both of the two-out-of-three results came from a blinded G&P reading.

In summary, regardless of match or discrepancy between clinical and research reads, the benchmark always included a blinded interpretation with G&P. This is important because G&P is the widely accepted method though intraobserver variability can be expected.

### Timing and reporting

Interpretation-report cycle time was defined as the time interval beginning with the study being opened on PACS and ending with the corrected report being signed in our reporting application (Powerscribe, Nuance Communications, Inc., Burlington, MA). The cycle included reviewing the study, referencing a bone age resource (G&P or DBAC), and creating and editing the report in Powerscribe. The faculty radiologists signed reports in the same reporting application (i.e., Powerscribe) regardless of assigned atlas; however, the DBAC workflow included an additional step whereby the first report draft was initiated by DBAC and copied into Powerscribe for editing and signature. While Powerscribe offers automated structured report templates and auto-import of some patient information regardless of use of G&P or DBAC, the purpose of the extra DBAC step was to use context sharing to automatically launch a proposed matching standard by age and gender, automate look-up of standard deviation value by age and gender, auto-calculate the number of standard deviations the patient is from the mean to aid final maturity assessment by the radiologist, and populate this information into a structured report.

### Subjective workflow experience

After completing all research interpretations in this study, each radiologist completed a Likert survey measuring subjective preferences between methods in the following nine categories: “image quality to include fine bone detail available in the images,” “utility of text and/or arrows in providing aid to interpretation,” “accuracy,” “efficiency,” “ease of use,” “confidence in my calculations,” “confidence in my overall result,” “resident checkout of bone age studies (from experience using both methods clinically with residents),” and “overall experience when reading bone age studies without a resident.” Each category included no further information beyond the above category names, and readers were asked to choose one of the following subjective responses: strongly prefer G&P, prefer G&P, no preference, prefer DBAC, or strongly prefer DBAC.

### Statistical analysis

#### Intraobserver agreement and agreement with benchmark

Intraobserver agreement for both bone age standard determination and overall maturity assessment (i.e., normal, delayed, or advanced) was summarized as the percentage of reads in which old and new determinations agreed. Intraobserver or intrarater agreement represents the degree of agreement for a single reader performing repeated bone age interpretations on the same study, i.e., how often does the reader agree with himself or herself when re-reading the same study? The intention was to compare how likely the reader was to agree with his/her original clinical interpretation with G&P with one method or the other. Since DBAC is based upon G&P, and G&P has known intraobserver variability, one might expect similar rates of intraobserver variability. Benchmark agreement represents the degree of agreement for each method to the benchmark result. Benchmark agreement allows a more reliable assessment of DBAC performance than comparing to only a single G&P result because of intraobserver variability intrinsic to G&P. Comparisons of agreement were conducted by way of Fisher’s exact tests, and *p* ≤ 0.05 was utilized to determine statistical significance.

#### Interpretation-report cycle time

The interpretation-reporting times were analyzed on the natural logarithmic scale via a linear mixed model (LMM). Hypothesis testing was conducted by way of a linear contrast of the LMM least squares means using *p* ≤ 0.05 to determine statistical significance. Since the data were analyzed on the natural logarithmic scale, the results were transformed to the geometric mean.

#### Report typographical/transcription errors

Report error rates were compared between the two methods via a paired random permutation test. To clarify the rationale for including this metric, we emphasize that DBAC produces a standardized report based upon the standard selection by the reader. Report generation is made possible because the gender, date of birth, and date of study were already made available to DBAC by electronic context sharing. The digital atlas looks up the appropriate standard deviation and completes basic calculations. The thought is that this semiautomated report may contain fewer textual errors than one generated by a radiologist.

#### Subjective workflow experience

An exact binomial test was utilized to test whether the readers’ responses to the survey questions could have occurred simply by chance if the reader did not prefer one bone age assessment method over the other.

## Results

### Intraobserver agreement

When the combined results of both readers were analyzed, intraobserver agreement between old and new G&P reads (*n* = 48) for bone age standard (i.e., selection of the same reference standard) and overall assessment of skeletal maturity (i.e., normal, advanced, or delayed) was found to be 85% [95% CI: (72, 94%)] and 94% [95% CI: (83, 99%)], respectively (Table 2). Intraobserver agreement between old G&P and new DBAC reads (*n* = 48) for bone age standard was 81% [95% CI: (67, 91%)] and for skeletal maturity was 96% [95% CI: (86, 99%)]. Intraobserver agreement for old G&P versus new G&P reads was not significantly different from old G&P versus new DBAC reads for both skeletal age (P = 0.785) and overall skeletal maturity (P = 1.000) assignments.

**Table 2:** Summary of intraobserver agreement for bone age standard selection and overall impression of skeletal maturity determined by DBAC or G&P methods for both readers combined

	Method	Agreement n/N (%)	95% CI	P value
Standard selection	G&P	41/48 (85)	[72, 94]	0.785
	DBAC	39/48 (81)	[67, 91]	
Overall skeletal maturity	G&P	45/48 (94)	[83, 99]	1.000
	DBAC	46/48 (96)	[86, 99]	

### Discrepant results

For 7 of 48 (15%) G&P study reads and 9 of 48 (19%) DBAC study reads, the bone age standard selection disagreed with the initial clinical standard selection and thus required tie-break reads with G&P (Table 2). For these examinations with discrepancies in standard selection, five of seven (71%) study reads with the G&P method and nine of nine (100%) study reads with DBAC agreed with the G&P tie-break read. Both discrepant G&P research reads were within one bone age standard of the benchmark.

With regard to overall skeletal maturity assignment (i.e., normal, delayed, or advanced), 3 of 48 (6%) G&P study reads and 2 of 48 (4%) DBAC study reads disagreed with the clinical maturity assignment and required tie-break reads with G&P (Table 2). In these discrepant cases, the study skeletal maturity assignment agreed with the tie-break G&P read for one of three (33%) G&P study reads and two of two (100%) DBAC study reads.

### Agreement with the benchmark

For the 96 included bone age examinations, there was perfect agreement [100%; 95% CI: (96, 100%)] between DBAC study reads and the benchmark for both bone age determination and overall skeletal maturity (Table 3). There was excellent agreement [96%; 95% CI: (90, 99%)] between G&P study reads and the benchmark for both bone age determination and overall skeletal maturity. There was no significant difference between agreement with the benchmark for DBAC compared with G&P for either bone age standard selection (*P* = 0.495) or overall skeletal maturity determination (*P* = 0.495).

**Table 3:** Summary of agreement with the benchmark for bone age standard selection and overall impression of skeletal maturity as determined by DBAC or G&P methods for both readers combined

	Method	Agreement n/N (%)	95% CI	P value
Standard selection	G&P	46/48 (96)	[90, 99]	0.495
	DBAC	48/48 (100)	[96, 100]	
Overall skeletal maturity	G&P	46/48 (96)	[90, 99]	0.495
	DBAC	48/48 (100)	[96, 100]	

### Interpretation-report cycle time

**Fig. 2:**
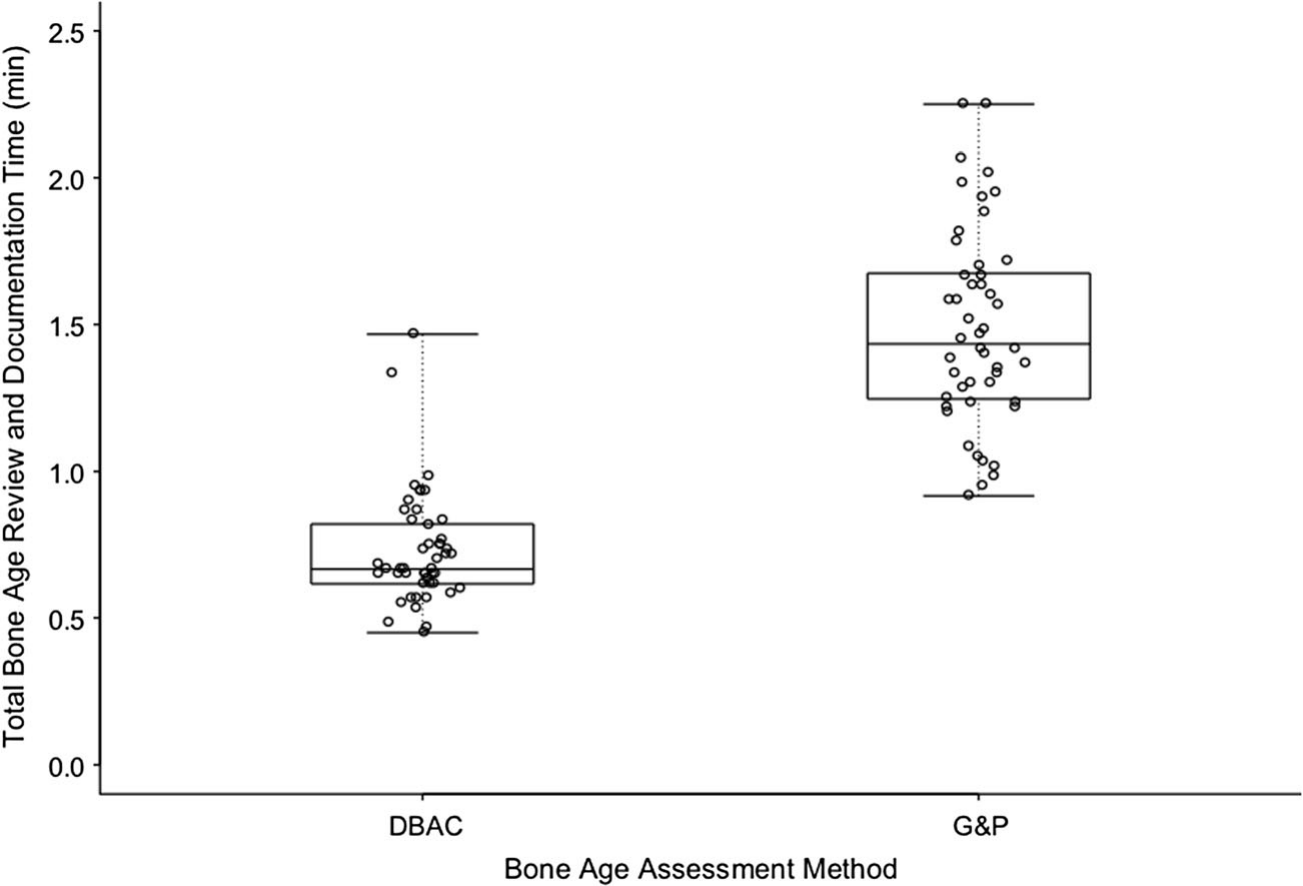
Box and whisker plot demonstrating the distribution of the bone age interpretation-report cycle times in minutes between the integrated electronic method of the Digital Bone Age Companion (DBAC) and the method of Greulich and Pyle (G&P) for the two faculty pediatric radiologists combined. Interpretation-report cycle time was defined as the time interval beginning with the study loading on PACS and ending with signing of the corrected report. Each circle represents an interpretation-report cycle time; the boxes represent the middle 50%; the lines within the boxes represent the mean; the superior- and inferior-most lines represent the maximum and minimum times, respectively

The geometric mean (GM) interpretation-report cycle time for DBAC [0.71 min; 95% CI: (0.40, 1.26 min)] method was 49% of that with G&P [GM = 1.45 min; 95% CI: (0.81, 2.59 min)] (Table 4) (Fig. 2), and this observed difference was statistically significant (*p* < 0.001).

**Table 4:** Summary statistics for interpretation-report cycle time (all times in minutes) and report error frequencies by review method for both radiologists combined

Method	n	Mean	SD	Geometric mean	Median	25th Percentile	75th Percentile	Min	Max
G&P	48	1.49	0.34	1.45	1.43	1.25	1.68	0.92	2.25
DBAC	48	0.73	0.19	0.71	0.67	0.62	0.82	0.45	1.47
Report errors?									
Method	Yes	No	Typographical/speech recognition error frequency (%)	95% CI					
G&P	11	37	22.9	[12.0, 37.3%]					
DBAC	0	48	0	[0, 7.4%]

### Report typographical/transcription errors

Among the 48 studies reported using the G&P method, there were a total of 11 typographical or speech recognition errors [22.9% error rate; 95% CI: (12.0, 37.3%)] as compared to zero [0% error rate; 95% CI: (0, 7.4%)] errors among the 48 studies reported with DBAC (Table 4). This observed difference in error rates was statistically significant (*p* = 0.002).

### Subjective workflow experience

One of the two pediatric radiologists indicated a “strong preference” for DBAC with regard to all nine of the Likert items listed in our Methods. The other radiologist indicated a “strong preference” for DBAC for all items except for “confidence in my overall result,” “image quality to include fine bone detail available in the images,” and “accuracy.” This radiologist indicated “no preference” between DBAC and G&P with regard to “image quality” and a “preference” for DBAC with regard to both “confidence in overall result” and subjective assessment of “accuracy.” In total, 17 of the 18 survey responses [94.4%; 95% CI: (72.7, 99.8%)] indicated a preference or strong preference for DBAC over G&P (*p* < 0.001).

## Discussion

G&P remains in widespread use for skeletal age determination by radiologists, pediatric orthopaedists, and pediatric endocrinologists [13–17] despite requiring a manual process that is more time-consuming than many other plain radiographic examinations. A new atlas based upon G&P could be a suitable alternative if it generated similar results, while updating to an electronic format might better integrate into contemporary workflow.

We found that two experienced faculty pediatric radiologists reached similar bone age assessments using G&P and DBAC, with the intraobserver agreement documented in this study comparable to though slightly less than what has been reported in the literature [2, 13, 18]. Our two pediatric radiologists realized time savings with DBAC, reporting studies in 49% of the time it took them when using the G&P atlas, despite their extensive experience with the older format.

Additionally, the bone age-specific report generation step in DBAC led to elimination of typographical and speech recognition reporting errors for our two radiologists, though other radiologists might have a different experience. While none of the reporting errors in our study were likely to be clinically significant, they did create a less cosmetic result. Still, reporting errors have the potential to be clinically significant if they are particularly unfortunate ones that are not identified and corrected. The use of patient and bone-age specific context integration to ensure that radiologists always (1) view bone age standards of the correct gender, (2) utilize the correct standard deviation value by gender and age, and (3) make correct calculations should theoretically reduce the likelihood of a clinically significant reporting error that might occur with a manual approach across a large number of studies interpreted by radiologists in different practice scenarios. Our study lacked power to detect a potential difference in clinically significant errors between methods because of the very low frequency of these types of errors among subspecialty faculty pediatric radiologists when interpreting bone age studies.

It is worth noting a potential source of bias with the use of DBAC when using automated context integration by age and gender. Berst et al. found that “observers are more likely to interpret the radiograph as showing normal findings when chronologic age is known than if the interpretation [of bone age studies] is performed with the observer unaware of the chronologic age” [18]. In our current study, our readers were automatically presented with a standard of similar age, which introduces exposure to this potential bias reported by Berst et al. Regardless of this potential effect, our study did not find a significant difference in performance between the use of G&P and DBAC with regard to skeletal maturity assessment.

Another potential source of bias in this report is that two of the five co-authors (CG, PB) are also co-authors of DBAC though only one (CG) has a financial conflict of interest. To limit this source of potentially significant bias, the study design excluded these two co-authors at key steps, most notably including image interpretation and statistical analysis. Still, although these two co-authors are not pediatric radiologists and one is a trainee, there is theoretical potential for bias because of peer influence, so for some level of reassurance it is also worth noting that the five co-authors are at three different institutions and none are currently in the same division at the same institution.

A few atlases containing left hand radiographic standards have been developed and marketed as alternatives to G&P [8–10]; however, there is a paucity of peer reviewed data on whether these alternatives yield similar results to G&P or whether they might improve productivity, and DBAC specifically has not been previously studied. This current study provides objective data supporting both the validity and usability of DBAC as a commercially available alternative to G&P for bone age assessment.

We believe that our study is limited by small sample size and the experience of only two academic pediatric radiologists. Their performance may not be representative of the general population of bone age interpreters. This issue of generalizability could be addressed with more broad future studies investigating DBAC. Additionally, we reported that our pediatric radiologists preferred their subjective experiences with the digital method and others may not reach similar conclusions following a similar experience. We debated including these subjective results; however, we felt there was some value in conveying some experience compared with none at all.

The fact that this study did not assess interobserver variability could be considered a limitation. However, the focus of our work was on intraobserver variability, as our purpose was to test whether a reader would achieve similar bone age interpretations whether using a new digital atlas or the widely accepted G&P atlas.

While it has been questioned if G&P may no longer be a valid tool for assessing skeletal maturity in modern and diverse populations because the atlas is based upon a study of predominantly Caucasian, affluent children in the 1930s [14, 15], this argument has generally not been born out in the literature [14, 16, 17, 19]. Thus, we believe there is rationale for a new bone age atlas to be based upon and compared to the long-standing G&P atlas.

## Conclusion

In this study, radiologists highly experienced with the method of Greulich and Pyle reported bone age studies in half the time and with elimination of reporting errors with a new alternative digital atlas, which also garnered their user preferences. DBAC, which itself is based upon the G&P atlas, may provide a satisfactory alternative to G&P for skeletal age determination.
